# (2-Anilino-4-methyl­thia­zol-5-yl)(4-chloro­phen­yl)methanone

**DOI:** 10.1107/S1600536811053463

**Published:** 2011-12-17

**Authors:** Jing-Jing Liu, Ren-Lin Zheng, Sheng-Yong Yang, Yong-Mei Xie

**Affiliations:** a State Key Laboratory of Biotherapy and Cancer Center, West China Hospital, West China Medical School, Sichuan University, Chengdu 610041, People’s Republic of China

## Abstract

The title compound, C_17_H_13_ClN_2_OS, crystallizes with three independent mol­ecules (*A*, *B* and *C*) in the asymmetric unit which differ slightly in their conformations. In mol­ecule *A*, the thiazole ring makes dihedral angles of 27.44 (14) and 66.05 (6)° with the phenyl and chloro­benzene rings. In mol­ecule *B*, the respective angles are 29.09 (10) and 47.63 (9)°, while values of 25.67 (11) and 51.01 (7)° are observed in mol­ecule *C*. In the crystal, N—H⋯N and N—H⋯O hydrogen bonds generate a three-dimensional network structure.

## Related literature

For the biological activity and synthesis of phen­yl(thia­zol-5-yl)methanone derivatives, see: Moisés *et al.* (2000 ▶).
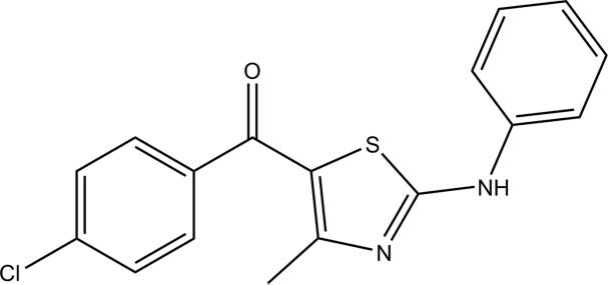


## Experimental

### 

#### Crystal data

C_17_H_13_ClN_2_OS
*M* *_r_* = 328.80
Monoclinic, 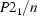

*a* = 22.8350 (6) Å
*b* = 8.0587 (2) Å
*c* = 25.3653 (6) Å
β = 90.900 (2)°
*V* = 4667.1 (2) Å^3^
*Z* = 12
Mo *K*α radiation
μ = 0.38 mm^−1^
*T* = 293 K
0.40 × 0.18 × 0.12 mm

#### Data collection

Agilent Xcalibur Eos diffractometer
Absorption correction: multi-scan (*CrysAlis PRO*; Agilent, 2011 ▶) *T*_min_ = 0.909, *T*_max_ = 1.000
18535 measured reflections
8224 independent reflections
5819 reflections with *I* > 2σ(*I*)
*R*_int_ = 0.020

#### Refinement

*R*[*F* ^2^ > 2σ(*F* ^2^)] = 0.047
*wR*(*F* ^2^) = 0.110
*S* = 1.02
8224 reflections
598 parameters
H-atom parameters constrained
Δρ_max_ = 0.31 e Å^−3^
Δρ_min_ = −0.29 e Å^−3^

### 

Data collection: *CrysAlis PRO* (Agilent, 2011 ▶); cell refinement: *CrysAlis PRO*; data reduction: *CrysAlis PRO*; program(s) used to solve structure: *SHELXS97* (Sheldrick, 2008 ▶); program(s) used to refine structure: *SHELXL97* (Sheldrick, 2008 ▶); molecular graphics: *OLEX2* (Dolomanov *et al.*, 2009 ▶) and *Mercury* (Macrae *et al.*, 2006 ▶); software used to prepare material for publication: *OLEX2*.

## Supplementary Material

Crystal structure: contains datablock(s) global, I. DOI: 10.1107/S1600536811053463/im2345sup1.cif

Structure factors: contains datablock(s) I. DOI: 10.1107/S1600536811053463/im2345Isup2.hkl

Supplementary material file. DOI: 10.1107/S1600536811053463/im2345Isup3.cml

Additional supplementary materials:  crystallographic information; 3D view; checkCIF report

## Figures and Tables

**Table 1:** Hydrogen-bond geometry (Å, °)

*D*—H⋯*A*	*D*—H	H⋯*A*	*D*⋯*A*	*D*—H⋯*A*
N2*A*—H2*A*⋯O1*C*^i^	0.86	2.09	2.933 (3)	167
N2*B*—H2*B*⋯N1*C*^ii^	0.86	2.11	2.962 (3)	170
N2*C*—H2*C*⋯N1*B*^iii^	0.86	2.17	2.995 (3)	162

Symmetry codes: (i) 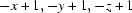; (ii) 
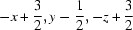
; (iii) 
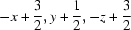
.

**Fig. 1.**
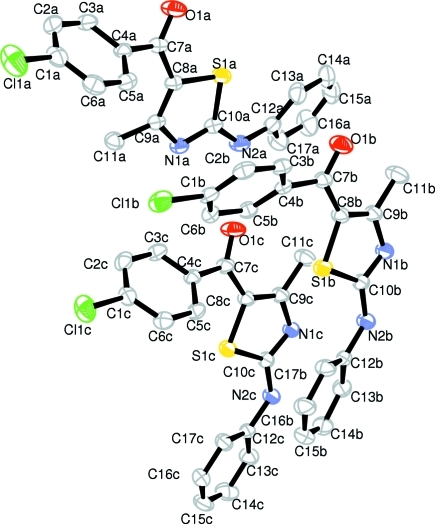
Molecular structure of the title compound with displacement ellipsoids drawn at the 30% probability level.

**Fig. 2.**
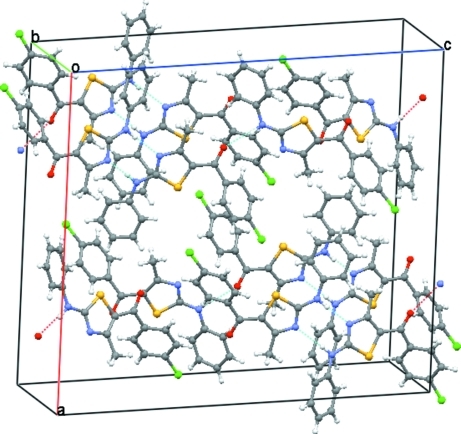
Packing diagram of the title compound. Hydrogen bonds are shown as blue dotted lines.

## supplementary crystallographic information

### Comment

(4-chlorophenyl)(4-methyl-2-(phenylamino)thiazol-5-yl)methanone derivatives are of great importance owing to their wide biological properties. The title compound is one of the key products in our synthetic investigations of antitumor drugs. We report here its crystal structure.

The title compound, C_17_H_13_ClN_2_OS, crystallizes with three independent molecules (A, B and C) per asymmetric unit. The independent molecules differ slightly in their conformations. In molecule A, the triazole ring makes dihedral angles of 27.44 (14)° and 66.05 (6)° with the phenyl and chlorobenzene rings. In molecule B the respective angles are 29.09 (10)° and 47.63 (9)°, while values of 25.67 (11)° and 51.01 (7)° are observed in molecule C. The molecules show intermolecular N2B—H2B···N1C, N2C—H2C···N1B and N2A—H2A···O1C hydrogen bonds. These bonds together with non-classical hydrogen bonds produce the oberved three dimensional network structure.

### Experimental

A suspension of acetimidamide hydrochloride (378.0 mg, 4.0 mmol) in THF (10.0 ml) was added to aqueous sodium hydroxide (1.6 ml, 2.5 N) and phenyl isothiocyanate (546.1 mg, 4.0 mmol) at 0°. The reaction mixture was stirred for 1–2 h at this temperature. It was then diluted with ethyl acetate (4.0 ml), the organic phase was washed with brine. Evaporation of the solvent in vacuo gave the *N*-(phenylcarbamothioyl)acetimidamide as solid.

*N*-(phenylcarbamothioyl)acetimidamide (386.5 mg, 2.0 mmol) was added to a solution of 2-bromo-1-(4-chlorophenyl)ethanone (524.2 mg, 2.2 mmol) in acetone containing triethylamine (0.28 ml, 2.0 mmol) at room temperature. After being stirred for 5–8 h, ethyl acetate was added, the organic phase was washed twice with brineand was afterwards evaporated *in vacuo*. The residue was recrystallized from ethanol to give the title compound as a light yellow solid (364.7 mg, 55.6% yield). Crystals suitable for X-ray analysis were obtained by slow evaporation from a solution of dichloromethane.

### Refinement

All H atoms were positioned geometrically and refined as rinding (C—H = 0.93–0.96 Å, N–H =0.86 Å) and with *U*_iso_(H) = 1.2*U*_eq_(C) or 1.5*U*_eq_(C) for methyl H atoms.

### Crystal data

**Table:**

C_17_H_13_ClN_2_OS	*F*(000) = 2040
*M**_r_* = 328.80	*D*_x_ = 1.404 Mg m^−^^3^
Monoclinic, *P*2_1_/*n*	Mo *K*α radiation, λ = 0.7107 Å
*a* = 22.8350 (6) Å	Cell parameters from 3809 reflections
*b* = 8.0587 (2) Å	θ = 3.0–29.2°
*c* = 25.3653 (6) Å	µ = 0.38 mm^−^^1^
β = 90.900 (2)°	*T* = 293 K
*V* = 4667.1 (2) Å^3^	Block, yellow
*Z* = 12	0.40 × 0.18 × 0.12 mm

### Data collection

**Table:**

Agilent Xcalibur Eos diffractometer	8224 independent reflections
Radiation source: Enhance (Mo) X-ray Source	5819 reflections with *I* > 2σ(*I*)
graphite	*R*_int_ = 0.020
Detector resolution: 16.0874 pixels mm^-1^	θ_max_ = 25.0°, θ_min_ = 3.0°
ω scans	*h* = −27→25
Absorption correction: multi-scan (*CrysAlis PRO*; Agilent, 2011)	*k* = −9→9
*T*_min_ = 0.909, *T*_max_ = 1.000	*l* = −20→30
18535 measured reflections	

### Refinement

**Table:**

Refinement on *F*^2^	Primary atom site location: structure-invariant direct methods
Least-squares matrix: full	Secondary atom site location: difference Fourier map
*R*[*F*^2^ > 2σ(*F*^2^)] = 0.047	Hydrogen site location: inferred from neighbouring sites
*wR*(*F*^2^) = 0.110	H-atom parameters constrained
*S* = 1.02	*w* = 1/[σ^2^(*F*_o_^2^) + (0.0402*P*)^2^ + 1.7472*P*] where *P* = (*F*_o_^2^ + 2*F*_c_^2^)/3
8224 reflections	(Δ/σ)_max_ = 0.001
598 parameters	Δρ_max_ = 0.31 e Å^−^^3^
0 restraints	Δρ_min_ = −0.29 e Å^−^^3^

### Special details

**Table:**

Experimental. CrysAlisPro, Agilent Technologies, Version 1.171.35.11 (release 16-05-2011 CrysAlis171 .NET) (compiled May 16 2011,17:55:39) Empirical absorption correction using spherical harmonics, implemented in SCALE3 ABSPACK scaling algorithm.
Geometry. All e.s.d.'s (except the e.s.d. in the dihedral angle between two l.s. planes) are estimated using the full covariance matrix. The cell e.s.d.'s are taken into account individually in the estimation of e.s.d.'s in distances, angles and torsion angles; correlations between e.s.d.'s in cell parameters are only used when they are defined by crystal symmetry. An approximate (isotropic) treatment of cell e.s.d.'s is used for estimating e.s.d.'s involving l.s. planes.
Refinement. Refinement of *F*^2^ against ALL reflections. The weighted *R*-factor *wR* and goodness of fit *S* are based on *F*^2^, conventional *R*-factors *R* are based on *F*, with *F* set to zero for negative *F*^2^. The threshold expression of *F*^2^ > σ(*F*^2^) is used only for calculating *R*-factors(gt) *etc*. and is not relevant to the choice of reflections for refinement. *R*-factors based on *F*^2^ are statistically about twice as large as those based on *F*, and *R*- factors based on ALL data will be even larger.

### Fractional atomic coordinates and isotropic or equivalent isotropic displacement parameters (Å^2^)

**Table:**

	*x*	*y*	*z*	*U*_iso_*/*U*_eq_	
Cl1A	0.45569 (5)	−0.04260 (15)	0.88138 (4)	0.1191 (4)	
Cl1B	0.56439 (4)	0.16982 (13)	0.35711 (3)	0.0837 (3)	
Cl1C	0.45183 (4)	0.73846 (13)	0.44041 (4)	0.0933 (3)	
S1A	0.19363 (3)	0.11756 (9)	0.64486 (3)	0.05524 (19)	
S1B	0.70318 (3)	0.14376 (10)	0.59119 (2)	0.0566 (2)	
S1C	0.60624 (3)	0.59963 (9)	0.65667 (2)	0.05367 (19)	
O1A	0.20043 (8)	0.0709 (3)	0.75853 (8)	0.0772 (6)	
O1B	0.81951 (9)	0.2484 (3)	0.48315 (8)	0.0805 (7)	
O1C	0.71946 (8)	0.6536 (3)	0.54361 (7)	0.0799 (7)	
N1A	0.28641 (9)	0.2498 (3)	0.60629 (8)	0.0497 (5)	
N1B	0.79856 (9)	0.1405 (3)	0.64667 (8)	0.0539 (6)	
N1C	0.70259 (9)	0.6152 (3)	0.71061 (7)	0.0497 (5)	
N2A	0.21002 (10)	0.2382 (3)	0.54573 (9)	0.0616 (6)	
H2A	0.2354	0.2638	0.5224	0.074*	
N2B	0.71638 (9)	0.0969 (3)	0.69726 (8)	0.0562 (6)	
H2B	0.7404	0.0897	0.7236	0.067*	
N2C	0.62296 (9)	0.5496 (3)	0.76210 (8)	0.0546 (6)	
H2C	0.6473	0.5522	0.7883	0.065*	
C1A	0.39626 (14)	−0.0021 (4)	0.84015 (13)	0.0691 (9)	
C1B	0.62500 (12)	0.1768 (4)	0.39942 (9)	0.0528 (7)	
C1C	0.51593 (12)	0.7180 (4)	0.47725 (11)	0.0560 (7)	
C2A	0.35340 (16)	0.1049 (4)	0.85619 (12)	0.0743 (9)	
H2AA	0.3561	0.1564	0.8889	0.089*	
C2B	0.66543 (13)	0.3021 (4)	0.39410 (10)	0.0574 (7)	
H2BA	0.6609	0.3811	0.3676	0.069*	
C2C	0.56031 (13)	0.6191 (4)	0.45884 (11)	0.0619 (8)	
H2CA	0.5560	0.5629	0.4270	0.074*	
C3A	0.30621 (13)	0.1348 (4)	0.82294 (11)	0.0632 (8)	
H3A	0.2766	0.2061	0.8336	0.076*	
C3B	0.71246 (12)	0.3092 (3)	0.42841 (10)	0.0525 (7)	
H3B	0.7398	0.3938	0.4250	0.063*	
C3C	0.61108 (13)	0.6047 (4)	0.48832 (10)	0.0591 (7)	
H3C	0.6417	0.5405	0.4757	0.071*	
C4A	0.30231 (11)	0.0602 (3)	0.77388 (9)	0.0479 (6)	
C4B	0.71992 (11)	0.1924 (3)	0.46808 (9)	0.0438 (6)	
C4C	0.61742 (11)	0.6842 (3)	0.53646 (9)	0.0460 (6)	
C5A	0.34582 (12)	−0.0468 (3)	0.75922 (11)	0.0555 (7)	
H5A	0.3435	−0.0982	0.7264	0.067*	
C5B	0.67921 (12)	0.0659 (3)	0.47177 (9)	0.0498 (7)	
H5B	0.6838	−0.0144	0.4978	0.060*	
C5C	0.57239 (12)	0.7849 (3)	0.55357 (10)	0.0517 (7)	
H5C	0.5763	0.8414	0.5854	0.062*	
C6A	0.39279 (13)	−0.0794 (4)	0.79216 (13)	0.0648 (8)	
H6A	0.4218	−0.1531	0.7820	0.078*	
C6B	0.63198 (12)	0.0573 (3)	0.43738 (10)	0.0533 (7)	
H6B	0.6051	−0.0289	0.4399	0.064*	
C6C	0.52187 (12)	0.8019 (4)	0.52375 (10)	0.0570 (7)	
H6C	0.4919	0.8703	0.5353	0.068*	
C7A	0.24934 (12)	0.0888 (3)	0.73979 (10)	0.0524 (7)	
C7B	0.77314 (12)	0.2045 (3)	0.50252 (10)	0.0528 (7)	
C7C	0.67305 (12)	0.6613 (3)	0.56691 (10)	0.0529 (7)	
C8A	0.25605 (11)	0.1340 (3)	0.68496 (9)	0.0470 (6)	
C8B	0.76968 (11)	0.1694 (3)	0.55904 (10)	0.0518 (7)	
C8C	0.67092 (11)	0.6435 (3)	0.62384 (9)	0.0485 (6)	
C9A	0.30000 (11)	0.2089 (3)	0.65721 (10)	0.0464 (6)	
C9B	0.81427 (11)	0.1650 (3)	0.59538 (10)	0.0524 (7)	
C9C	0.71662 (11)	0.6482 (3)	0.65933 (10)	0.0509 (7)	
C10A	0.23223 (12)	0.2084 (3)	0.59454 (10)	0.0488 (6)	
C10B	0.74124 (11)	0.1273 (3)	0.65030 (9)	0.0483 (6)	
C10C	0.64570 (11)	0.5866 (3)	0.71482 (9)	0.0472 (6)	
C11A	0.35989 (12)	0.2554 (3)	0.67677 (11)	0.0579 (7)	
H11G	0.3741	0.3477	0.6567	0.087*	
H11H	0.3858	0.1627	0.6728	0.087*	
H11I	0.3582	0.2856	0.7133	0.087*	
C11B	0.87818 (12)	0.1840 (5)	0.58394 (12)	0.0799 (10)	
H11D	0.8994	0.2045	0.6162	0.120*	
H11E	0.8835	0.2755	0.5603	0.120*	
H11F	0.8924	0.0841	0.5680	0.120*	
C11C	0.77868 (11)	0.6850 (4)	0.64657 (11)	0.0691 (9)	
H11A	0.7981	0.7314	0.6770	0.104*	
H11B	0.7799	0.7629	0.6180	0.104*	
H11C	0.7980	0.5843	0.6365	0.104*	
C12A	0.15138 (13)	0.2336 (4)	0.52755 (11)	0.0563 (7)	
C12B	0.65685 (11)	0.0753 (3)	0.70944 (9)	0.0477 (6)	
C12C	0.56479 (11)	0.5072 (3)	0.77476 (10)	0.0493 (6)	
C13A	0.11009 (15)	0.1252 (4)	0.54626 (12)	0.0723 (9)	
H13A	0.1207	0.0453	0.5711	0.087*	
C13B	0.64422 (13)	−0.0198 (3)	0.75335 (10)	0.0593 (7)	
H13B	0.6742	−0.0717	0.7723	0.071*	
C13C	0.55710 (13)	0.4119 (4)	0.81895 (11)	0.0634 (8)	
H13C	0.5894	0.3761	0.8387	0.076*	
C14A	0.05266 (16)	0.1350 (5)	0.52815 (14)	0.0855 (10)	
H14A	0.0246	0.0644	0.5420	0.103*	
C14B	0.58682 (15)	−0.0370 (4)	0.76867 (12)	0.0720 (9)	
H14B	0.5784	−0.0993	0.7985	0.086*	
C14C	0.50092 (16)	0.3691 (4)	0.83404 (14)	0.0820 (10)	
H14C	0.4958	0.3042	0.8639	0.098*	
C15A	0.03690 (16)	0.2461 (5)	0.49055 (16)	0.0896 (11)	
H15A	−0.0017	0.2515	0.4785	0.107*	
C15B	0.54219 (14)	0.0358 (4)	0.74082 (13)	0.0767 (10)	
H15B	0.5037	0.0238	0.7516	0.092*	
C15C	0.45340 (14)	0.4209 (4)	0.80572 (13)	0.0707 (9)	
H15C	0.4159	0.3929	0.8164	0.085*	
C16A	0.07810 (17)	0.3499 (5)	0.47044 (15)	0.0968 (12)	
H16A	0.0677	0.4250	0.4441	0.116*	
C16B	0.55463 (13)	0.1259 (4)	0.69724 (12)	0.0739 (9)	
H16B	0.5242	0.1726	0.6774	0.089*	
C16C	0.46096 (13)	0.5143 (4)	0.76149 (12)	0.0671 (8)	
H16C	0.4285	0.5465	0.7414	0.080*	
C17A	0.13499 (15)	0.3443 (4)	0.48897 (13)	0.0785 (9)	
H17A	0.1627	0.4164	0.4752	0.094*	
C17B	0.61178 (12)	0.1492 (4)	0.68190 (11)	0.0607 (7)	
H17B	0.6197	0.2152	0.6528	0.073*	
C17C	0.51638 (12)	0.5616 (4)	0.74619 (11)	0.0579 (7)	
H17C	0.5211	0.6294	0.7169	0.069*	

### Atomic displacement parameters (Å^2^)

**Table:**

	*U*^11^	*U*^22^	*U*^33^	*U*^12^	*U*^13^	*U*^23^
Cl1A	0.0988 (7)	0.1507 (10)	0.1066 (8)	−0.0353 (7)	−0.0406 (6)	0.0599 (7)
Cl1B	0.0652 (5)	0.1387 (8)	0.0467 (4)	0.0200 (5)	−0.0105 (4)	−0.0027 (5)
Cl1C	0.0778 (6)	0.1217 (7)	0.0791 (6)	−0.0140 (5)	−0.0353 (5)	0.0216 (5)
S1A	0.0490 (4)	0.0690 (5)	0.0480 (4)	−0.0036 (4)	0.0088 (3)	0.0057 (3)
S1B	0.0427 (4)	0.0932 (5)	0.0337 (3)	−0.0020 (4)	−0.0028 (3)	0.0066 (3)
S1C	0.0447 (4)	0.0818 (5)	0.0344 (3)	−0.0090 (4)	−0.0047 (3)	0.0095 (3)
O1A	0.0567 (13)	0.1144 (17)	0.0611 (13)	0.0001 (12)	0.0183 (10)	0.0244 (12)
O1B	0.0618 (13)	0.1272 (19)	0.0527 (12)	−0.0209 (13)	0.0057 (10)	0.0213 (12)
O1C	0.0498 (12)	0.146 (2)	0.0439 (11)	0.0114 (13)	0.0108 (9)	0.0207 (12)
N1A	0.0490 (13)	0.0584 (13)	0.0419 (12)	0.0017 (11)	0.0076 (10)	0.0047 (10)
N1B	0.0414 (12)	0.0808 (16)	0.0392 (12)	−0.0052 (12)	−0.0025 (10)	0.0054 (11)
N1C	0.0442 (13)	0.0719 (15)	0.0328 (11)	−0.0044 (11)	−0.0020 (9)	0.0059 (10)
N2A	0.0534 (14)	0.0877 (17)	0.0438 (13)	0.0038 (13)	0.0073 (11)	0.0087 (12)
N2B	0.0432 (12)	0.0912 (17)	0.0341 (11)	−0.0055 (12)	−0.0040 (10)	0.0079 (11)
N2C	0.0438 (12)	0.0865 (16)	0.0331 (11)	−0.0080 (12)	−0.0051 (10)	0.0124 (11)
C1A	0.067 (2)	0.080 (2)	0.060 (2)	−0.0218 (18)	−0.0043 (17)	0.0289 (17)
C1B	0.0538 (16)	0.0727 (18)	0.0320 (14)	0.0139 (15)	0.0042 (12)	−0.0028 (13)
C1C	0.0577 (18)	0.0650 (18)	0.0452 (16)	−0.0121 (15)	−0.0080 (13)	0.0172 (14)
C2A	0.086 (2)	0.096 (2)	0.0413 (17)	−0.029 (2)	0.0067 (17)	−0.0022 (17)
C2B	0.070 (2)	0.0656 (18)	0.0364 (14)	0.0218 (17)	0.0053 (14)	0.0132 (13)
C2C	0.078 (2)	0.0686 (19)	0.0389 (15)	−0.0053 (17)	−0.0029 (15)	0.0020 (14)
C3A	0.068 (2)	0.074 (2)	0.0482 (17)	−0.0057 (17)	0.0206 (15)	−0.0009 (15)
C3B	0.0639 (18)	0.0537 (16)	0.0404 (15)	0.0042 (14)	0.0120 (13)	0.0065 (12)
C3C	0.0676 (19)	0.0705 (18)	0.0393 (15)	0.0115 (16)	0.0055 (14)	0.0020 (14)
C4A	0.0550 (16)	0.0514 (15)	0.0378 (14)	−0.0064 (13)	0.0138 (12)	0.0062 (12)
C4B	0.0510 (15)	0.0525 (15)	0.0282 (12)	0.0066 (13)	0.0068 (11)	0.0006 (11)
C4C	0.0505 (15)	0.0559 (15)	0.0317 (13)	−0.0014 (13)	0.0036 (11)	0.0092 (12)
C5A	0.0636 (18)	0.0562 (16)	0.0467 (16)	0.0003 (15)	0.0085 (14)	0.0005 (13)
C5B	0.0600 (17)	0.0544 (16)	0.0351 (14)	0.0028 (14)	0.0034 (12)	0.0088 (12)
C5C	0.0597 (17)	0.0595 (16)	0.0358 (14)	0.0040 (14)	0.0000 (13)	0.0031 (12)
C6A	0.0587 (19)	0.0608 (18)	0.075 (2)	0.0010 (15)	0.0096 (17)	0.0165 (16)
C6B	0.0573 (17)	0.0607 (17)	0.0419 (15)	−0.0040 (14)	0.0017 (13)	−0.0014 (13)
C6C	0.0543 (17)	0.0707 (18)	0.0460 (16)	0.0105 (15)	0.0004 (13)	0.0111 (14)
C7A	0.0536 (17)	0.0560 (16)	0.0479 (16)	0.0012 (14)	0.0132 (14)	0.0057 (13)
C7B	0.0538 (17)	0.0631 (17)	0.0416 (15)	−0.0058 (14)	0.0045 (13)	0.0065 (13)
C7C	0.0532 (16)	0.0668 (17)	0.0390 (14)	0.0019 (14)	0.0041 (13)	0.0102 (13)
C8A	0.0505 (15)	0.0498 (15)	0.0409 (14)	0.0001 (13)	0.0079 (12)	0.0037 (12)
C8B	0.0444 (15)	0.0723 (18)	0.0388 (14)	−0.0040 (14)	0.0020 (12)	0.0077 (13)
C8C	0.0453 (15)	0.0641 (17)	0.0361 (13)	−0.0015 (13)	0.0013 (11)	0.0099 (12)
C9A	0.0511 (16)	0.0455 (14)	0.0427 (14)	0.0017 (13)	0.0095 (12)	0.0005 (12)
C9B	0.0468 (15)	0.0698 (18)	0.0406 (15)	−0.0068 (14)	−0.0003 (12)	0.0056 (13)
C9C	0.0449 (15)	0.0636 (17)	0.0443 (15)	−0.0001 (14)	0.0010 (12)	0.0081 (13)
C10A	0.0517 (17)	0.0517 (15)	0.0432 (15)	0.0065 (13)	0.0073 (12)	0.0021 (12)
C10B	0.0455 (15)	0.0653 (17)	0.0338 (13)	−0.0035 (14)	−0.0030 (11)	0.0038 (12)
C10C	0.0445 (15)	0.0604 (16)	0.0364 (14)	−0.0013 (13)	−0.0049 (11)	0.0059 (12)
C11A	0.0573 (18)	0.0618 (17)	0.0546 (17)	−0.0069 (15)	0.0053 (14)	0.0079 (14)
C11B	0.0435 (17)	0.137 (3)	0.0593 (19)	−0.0101 (19)	0.0020 (14)	0.0202 (19)
C11C	0.0416 (16)	0.109 (2)	0.0566 (18)	−0.0021 (16)	−0.0001 (13)	0.0177 (17)
C12A	0.0599 (18)	0.0638 (18)	0.0453 (16)	0.0028 (16)	0.0004 (14)	−0.0076 (14)
C12B	0.0464 (15)	0.0611 (16)	0.0354 (14)	−0.0049 (13)	−0.0008 (12)	−0.0052 (12)
C12C	0.0479 (16)	0.0598 (16)	0.0401 (14)	−0.0063 (13)	0.0023 (12)	0.0003 (13)
C13A	0.083 (2)	0.072 (2)	0.0616 (19)	−0.0138 (19)	−0.0119 (17)	−0.0002 (16)
C13B	0.0643 (19)	0.0659 (18)	0.0476 (16)	−0.0031 (15)	−0.0001 (14)	0.0043 (14)
C13C	0.0631 (19)	0.075 (2)	0.0522 (17)	−0.0060 (16)	0.0051 (14)	0.0137 (15)
C14A	0.079 (2)	0.101 (3)	0.076 (2)	−0.031 (2)	−0.014 (2)	−0.013 (2)
C14B	0.080 (2)	0.078 (2)	0.0579 (19)	−0.0186 (19)	0.0162 (18)	0.0050 (16)
C14C	0.086 (3)	0.085 (2)	0.076 (2)	−0.013 (2)	0.028 (2)	0.0233 (19)
C15A	0.073 (2)	0.102 (3)	0.093 (3)	−0.009 (2)	−0.027 (2)	−0.013 (2)
C15B	0.0543 (19)	0.103 (3)	0.074 (2)	−0.0167 (19)	0.0163 (17)	−0.008 (2)
C15C	0.0528 (19)	0.076 (2)	0.084 (2)	−0.0141 (17)	0.0217 (17)	0.0027 (19)
C16A	0.089 (3)	0.101 (3)	0.099 (3)	−0.006 (2)	−0.034 (2)	0.024 (2)
C16B	0.0487 (18)	0.108 (3)	0.065 (2)	0.0086 (18)	0.0009 (15)	−0.0023 (19)
C16C	0.0509 (18)	0.077 (2)	0.073 (2)	−0.0091 (16)	0.0009 (15)	−0.0079 (17)
C17A	0.069 (2)	0.091 (2)	0.075 (2)	−0.0071 (19)	−0.0120 (18)	0.0187 (19)
C17B	0.0511 (17)	0.084 (2)	0.0470 (16)	0.0031 (16)	0.0039 (13)	0.0096 (15)
C17C	0.0475 (16)	0.0748 (19)	0.0512 (17)	−0.0091 (15)	−0.0003 (13)	0.0057 (14)

### Geometric parameters (Å, °)

**Table:**

Cl1A—C1A	1.731 (3)	C5C—C6C	1.376 (3)
Cl1B—C1B	1.739 (3)	C6A—H6A	0.9300
Cl1C—C1C	1.732 (3)	C6B—H6B	0.9300
S1A—C8A	1.743 (3)	C6C—H6C	0.9300
S1A—C10A	1.725 (3)	C7A—C8A	1.448 (3)
S1B—C8B	1.747 (3)	C7B—C8B	1.465 (3)
S1B—C10B	1.726 (2)	C7C—C8C	1.453 (3)
S1C—C8C	1.743 (2)	C8A—C9A	1.375 (3)
S1C—C10C	1.720 (2)	C8B—C9B	1.363 (3)
O1A—C7A	1.229 (3)	C8C—C9C	1.368 (3)
O1B—C7B	1.226 (3)	C9A—C11A	1.495 (3)
O1C—C7C	1.223 (3)	C9B—C11B	1.500 (3)
N1A—C9A	1.364 (3)	C9C—C11C	1.489 (3)
N1A—C10A	1.311 (3)	C11A—H11G	0.9600
N1B—C9B	1.369 (3)	C11A—H11H	0.9600
N1B—C10B	1.318 (3)	C11A—H11I	0.9600
N1C—C9C	1.370 (3)	C11B—H11D	0.9600
N1C—C10C	1.326 (3)	C11B—H11E	0.9600
N2A—H2A	0.8600	C11B—H11F	0.9600
N2A—C10A	1.352 (3)	C11C—H11A	0.9600
N2A—C12A	1.410 (3)	C11C—H11B	0.9600
N2B—H2B	0.8600	C11C—H11C	0.9600
N2B—C10B	1.350 (3)	C12A—C13A	1.375 (4)
N2B—C12B	1.410 (3)	C12A—C17A	1.372 (4)
N2C—H2C	0.8600	C12B—C13B	1.386 (3)
N2C—C10C	1.348 (3)	C12B—C17B	1.371 (3)
N2C—C12C	1.414 (3)	C12C—C13C	1.372 (4)
C1A—C2A	1.371 (4)	C12C—C17C	1.383 (4)
C1A—C6A	1.369 (4)	C13A—H13A	0.9300
C1B—C2B	1.377 (4)	C13A—C14A	1.385 (4)
C1B—C6B	1.369 (4)	C13B—H13B	0.9300
C1C—C2C	1.377 (4)	C13B—C14B	1.380 (4)
C1C—C6C	1.365 (4)	C13C—H13C	0.9300
C2A—H2AA	0.9300	C13C—C14C	1.388 (4)
C2A—C3A	1.379 (4)	C14A—H14A	0.9300
C2B—H2BA	0.9300	C14A—C15A	1.352 (5)
C2B—C3B	1.373 (4)	C14B—H14B	0.9300
C2C—H2CA	0.9300	C14B—C15B	1.364 (4)
C2C—C3C	1.375 (4)	C14C—H14C	0.9300
C3A—H3A	0.9300	C14C—C15C	1.358 (4)
C3A—C4A	1.384 (4)	C15A—H15A	0.9300
C3B—H3B	0.9300	C15A—C16A	1.364 (5)
C3B—C4B	1.386 (3)	C15B—H15B	0.9300
C3C—H3C	0.9300	C15B—C16B	1.356 (4)
C3C—C4C	1.385 (3)	C15C—H15C	0.9300
C4A—C5A	1.371 (3)	C15C—C16C	1.364 (4)
C4A—C7A	1.494 (4)	C16A—H16A	0.9300
C4B—C5B	1.384 (3)	C16A—C17A	1.375 (4)
C4B—C7B	1.489 (3)	C16B—H16B	0.9300
C4C—C5C	1.385 (3)	C16B—C17B	1.381 (4)
C4C—C7C	1.488 (3)	C16C—H16C	0.9300
C5A—H5A	0.9300	C16C—C17C	1.383 (4)
C5A—C6A	1.375 (4)	C17A—H17A	0.9300
C5B—H5B	0.9300	C17B—H17B	0.9300
C5B—C6B	1.378 (3)	C17C—H17C	0.9300
C5C—H5C	0.9300		
			
C10A—S1A—C8A	88.68 (12)	C8A—C9A—C11A	127.7 (2)
C10B—S1B—C8B	89.14 (12)	N1B—C9B—C11B	118.0 (2)
C10C—S1C—C8C	89.17 (12)	C8B—C9B—N1B	116.2 (2)
C10A—N1A—C9A	110.7 (2)	C8B—C9B—C11B	125.8 (2)
C10B—N1B—C9B	110.7 (2)	N1C—C9C—C11C	118.8 (2)
C10C—N1C—C9C	110.7 (2)	C8C—C9C—N1C	115.6 (2)
C10A—N2A—H2A	115.3	C8C—C9C—C11C	125.6 (2)
C10A—N2A—C12A	129.5 (2)	N1A—C10A—S1A	115.5 (2)
C12A—N2A—H2A	115.3	N1A—C10A—N2A	120.1 (2)
C10B—N2B—H2B	115.2	N2A—C10A—S1A	124.3 (2)
C10B—N2B—C12B	129.5 (2)	N1B—C10B—S1B	114.85 (18)
C12B—N2B—H2B	115.2	N1B—C10B—N2B	120.5 (2)
C10C—N2C—H2C	115.4	N2B—C10B—S1B	124.6 (2)
C10C—N2C—C12C	129.2 (2)	N1C—C10C—S1C	114.92 (18)
C12C—N2C—H2C	115.4	N1C—C10C—N2C	120.1 (2)
C2A—C1A—Cl1A	119.7 (3)	N2C—C10C—S1C	124.9 (2)
C6A—C1A—Cl1A	119.0 (3)	C9A—C11A—H11G	109.5
C6A—C1A—C2A	121.2 (3)	C9A—C11A—H11H	109.5
C2B—C1B—Cl1B	119.4 (2)	C9A—C11A—H11I	109.5
C6B—C1B—Cl1B	119.6 (2)	H11G—C11A—H11H	109.5
C6B—C1B—C2B	121.0 (3)	H11G—C11A—H11I	109.5
C2C—C1C—Cl1C	119.5 (2)	H11H—C11A—H11I	109.5
C6C—C1C—Cl1C	119.4 (2)	C9B—C11B—H11D	109.5
C6C—C1C—C2C	121.1 (3)	C9B—C11B—H11E	109.5
C1A—C2A—H2AA	120.6	C9B—C11B—H11F	109.5
C1A—C2A—C3A	118.9 (3)	H11D—C11B—H11E	109.5
C3A—C2A—H2AA	120.6	H11D—C11B—H11F	109.5
C1B—C2B—H2BA	120.4	H11E—C11B—H11F	109.5
C3B—C2B—C1B	119.2 (2)	C9C—C11C—H11A	109.5
C3B—C2B—H2BA	120.4	C9C—C11C—H11B	109.5
C1C—C2C—H2CA	120.5	C9C—C11C—H11C	109.5
C3C—C2C—C1C	118.9 (3)	H11A—C11C—H11B	109.5
C3C—C2C—H2CA	120.5	H11A—C11C—H11C	109.5
C2A—C3A—H3A	119.6	H11B—C11C—H11C	109.5
C2A—C3A—C4A	120.8 (3)	C13A—C12A—N2A	123.8 (3)
C4A—C3A—H3A	119.6	C17A—C12A—N2A	117.6 (3)
C2B—C3B—H3B	119.4	C17A—C12A—C13A	118.6 (3)
C2B—C3B—C4B	121.2 (3)	C13B—C12B—N2B	117.3 (2)
C4B—C3B—H3B	119.4	C17B—C12B—N2B	123.6 (2)
C2C—C3C—H3C	119.5	C17B—C12B—C13B	119.1 (2)
C2C—C3C—C4C	121.1 (3)	C13C—C12C—N2C	117.0 (2)
C4C—C3C—H3C	119.5	C13C—C12C—C17C	119.6 (3)
C3A—C4A—C7A	119.6 (2)	C17C—C12C—N2C	123.4 (2)
C5A—C4A—C3A	118.7 (3)	C12A—C13A—H13A	120.0
C5A—C4A—C7A	121.6 (2)	C12A—C13A—C14A	120.0 (3)
C3B—C4B—C7B	118.1 (2)	C14A—C13A—H13A	120.0
C5B—C4B—C3B	118.3 (2)	C12B—C13B—H13B	120.3
C5B—C4B—C7B	123.5 (2)	C14B—C13B—C12B	119.5 (3)
C3C—C4C—C5C	118.7 (2)	C14B—C13B—H13B	120.3
C3C—C4C—C7C	118.5 (2)	C12C—C13C—H13C	120.2
C5C—C4C—C7C	122.8 (2)	C12C—C13C—C14C	119.7 (3)
C4A—C5A—H5A	119.4	C14C—C13C—H13C	120.2
C4A—C5A—C6A	121.1 (3)	C13A—C14A—H14A	119.6
C6A—C5A—H5A	119.4	C15A—C14A—C13A	120.8 (3)
C4B—C5B—H5B	119.5	C15A—C14A—H14A	119.6
C6B—C5B—C4B	120.9 (2)	C13B—C14B—H14B	119.5
C6B—C5B—H5B	119.5	C15B—C14B—C13B	121.1 (3)
C4C—C5C—H5C	119.8	C15B—C14B—H14B	119.5
C6C—C5C—C4C	120.4 (2)	C13C—C14C—H14C	119.6
C6C—C5C—H5C	119.8	C15C—C14C—C13C	120.8 (3)
C1A—C6A—C5A	119.2 (3)	C15C—C14C—H14C	119.6
C1A—C6A—H6A	120.4	C14A—C15A—H15A	120.3
C5A—C6A—H6A	120.4	C14A—C15A—C16A	119.5 (3)
C1B—C6B—C5B	119.4 (3)	C16A—C15A—H15A	120.3
C1B—C6B—H6B	120.3	C14B—C15B—H15B	120.4
C5B—C6B—H6B	120.3	C16B—C15B—C14B	119.2 (3)
C1C—C6C—C5C	119.8 (3)	C16B—C15B—H15B	120.4
C1C—C6C—H6C	120.1	C14C—C15C—H15C	120.2
C5C—C6C—H6C	120.1	C14C—C15C—C16C	119.6 (3)
O1A—C7A—C4A	119.4 (2)	C16C—C15C—H15C	120.2
O1A—C7A—C8A	120.8 (3)	C15A—C16A—H16A	119.8
C8A—C7A—C4A	119.9 (2)	C15A—C16A—C17A	120.3 (3)
O1B—C7B—C4B	119.1 (2)	C17A—C16A—H16A	119.8
O1B—C7B—C8B	120.5 (3)	C15B—C16B—H16B	119.5
C8B—C7B—C4B	120.4 (2)	C15B—C16B—C17B	120.9 (3)
O1C—C7C—C4C	119.6 (2)	C17B—C16B—H16B	119.5
O1C—C7C—C8C	121.2 (2)	C15C—C16C—H16C	119.6
C8C—C7C—C4C	119.1 (2)	C15C—C16C—C17C	120.7 (3)
C7A—C8A—S1A	116.33 (19)	C17C—C16C—H16C	119.6
C9A—C8A—S1A	109.35 (18)	C12A—C17A—C16A	120.7 (3)
C9A—C8A—C7A	133.7 (3)	C12A—C17A—H17A	119.6
C7B—C8B—S1B	122.7 (2)	C16A—C17A—H17A	119.6
C9B—C8B—S1B	109.08 (18)	C12B—C17B—C16B	120.2 (3)
C9B—C8B—C7B	128.0 (2)	C12B—C17B—H17B	119.9
C7C—C8C—S1C	122.5 (2)	C16B—C17B—H17B	119.9
C9C—C8C—S1C	109.55 (18)	C12C—C17C—H17C	120.2
C9C—C8C—C7C	127.9 (2)	C16C—C17C—C12C	119.5 (3)
N1A—C9A—C8A	115.7 (2)	C16C—C17C—H17C	120.2
N1A—C9A—C11A	116.6 (2)		

### Hydrogen-bond geometry (Å, °)

**Table:**

*D*—H···*A*	*D*—H	H···*A*	*D*···*A*	*D*—H···*A*
N2A—H2A···O1C^i^	0.86	2.09	2.933 (3)	167.
N2B—H2B···N1C^ii^	0.86	2.11	2.962 (3)	170.
N2C—H2C···N1B^iii^	0.86	2.17	2.995 (3)	162.

Symmetry codes: (i) −*x*+1, −*y*+1, −*z*+1; (ii) −*x*+3/2, *y*−1/2, −*z*+3/2; (iii) −*x*+3/2, *y*+1/2, −*z*+3/2.
